# Synthetic Biology—High Time to Deliver?

**DOI:** 10.1021/acssynbio.3c00238

**Published:** 2023-06-16

**Authors:** Andrew D. Hanson, Víctor de Lorenzo

**Affiliations:** † Horticultural Sciences Department, University of Florida, Gainesville, Florida 32611, United States; ‡ Systems Biology Department, Centro Nacional de Biotecnología-CSIC, Campus de Cantoblanco, Madrid 28049, Spain

## Abstract

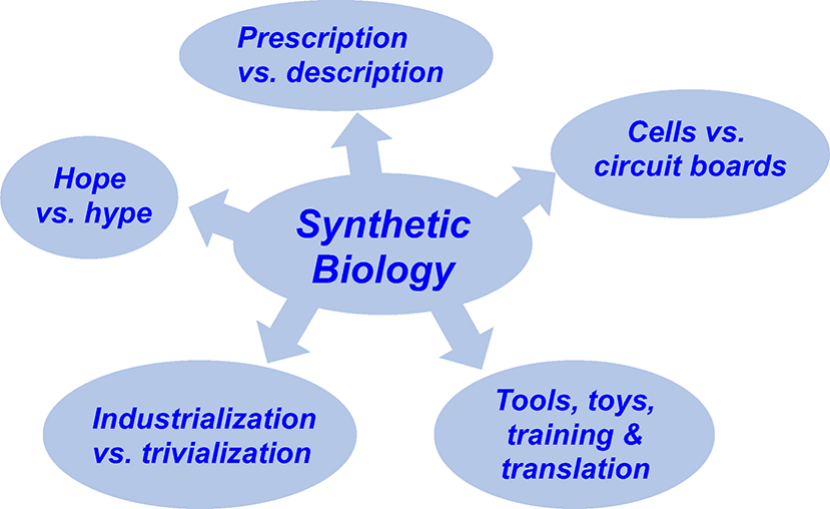

Synthetic biology (SynBio) has attracted like no other recent development the attention not only of Life Science researchers and engineers but also of intellectuals, technology think-tanks, and private and public investors. This is largely due to its promise to propel biotechnology beyond its traditional realms in medicine, agriculture, and environment toward new territories historically dominated by the chemical and manufacturing industries—but now claimed to be amenable to complete *biologization*. For this to happen, it is crucial for the field to remain true to its foundational engineering drive, which relies on mathematics and quantitative tools to construct practical solutions to real-world problems. This article highlights several SynBio themes that, in our view, come with somewhat precarious promises that need to be tackled. First, SynBio must critically examine whether enough basic information is available to enable the design or redesign of life processes and turn biology from a descriptive science into a prescriptive one. Second, unlike circuit boards, cells are built with soft matter and possess inherent abilities to mutate and evolve, even without external cues. Third, the field cannot be presented as the one technical solution to many grave world problems and so must avoid exaggerated claims and hype. Finally, SynBio should pay heed to public sensitivities and involve social science in its development and growth, and thus change the technology narrative from sheer domination of the living world to conversation and win-win partnership.

In the modern sense, the scientific and technical field universally known now as Synthetic Biology (SynBio) first took off a little more than 20 years ago in bacteria and yeast (Figure 1) and, although it soon reached plants, animals, and cell-free systems, it remains largely microbe-centric.^1−5^ Notwithstanding this constraint, nothing in biology since the double helix and recombinant DNA revolutions has captured more scientific, philosophical, government, and investor interest than SynBio.^6−8^ Nor has any other field generated such a vast speculative secondary literature on how it could transform everything from manufacturing, agriculture, and medicine to ecosystems, earth’s climate, and space travel, plus resurrect the Pleistocene paleofauna.^8−10^ Along the way, much has been promised, implicitly or explicitly. Here, we argue that a good deal of this promise may not be fulfilled unless SynBio stays anchored to its engineering foundations, i.e., applies science, math, and art to design and build workable solutions to real problems. We cover five SynBio themes fraught with easily breakable promises. We do this briefly, to spill as little SynBio ink^11^ as possible.

**Figure 1:**
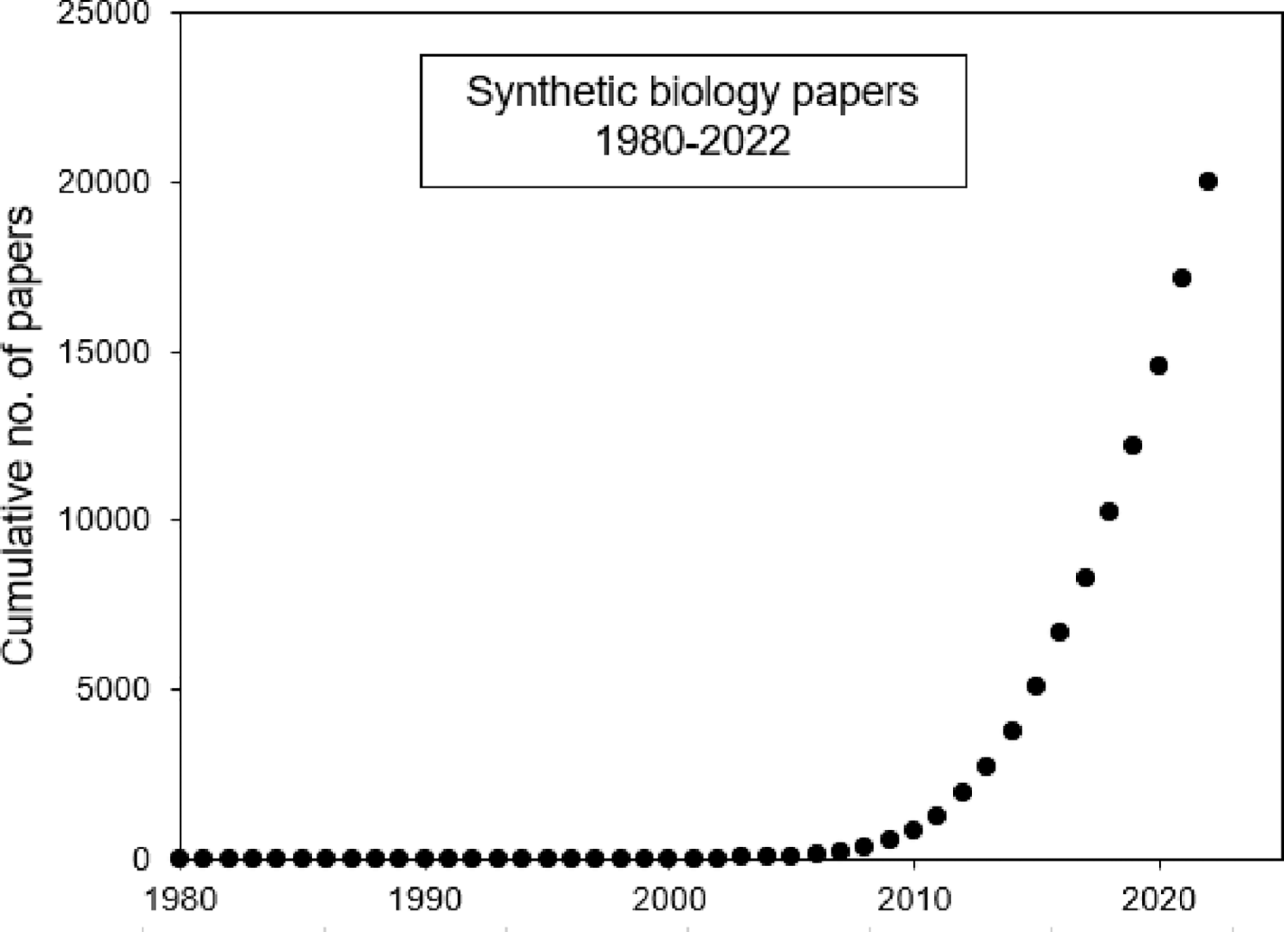
Explosive growth of the SynBio literature.

## Prescription versus Description

SynBio has promised to change biology from an essentially descriptive science into a prescriptive one where reliable functional predictions are made and implemented.^1,12^ The idea (“build to understand”) is that SynBio can help the biosciences to escape the vortex of ever-more-detailed-description that has trapped them for centuries, and enter a new phase of deeper, more actionable knowledge of life. Much conventional biological research tends to tunnel down on complexity and detail, describes these with each new toolset that becomes available, then moves on to describe another system. This traditional routine generates papers, trains PhDs, and builds careers, but often fails to produce information that is either profound or useful in improving the systems studied. This constitutes a contractual default–a broken promise–inasmuch as the societal *quid pro quo* for funding bioscience is the discovery of profound and useful things.^13^ To avoid being subverted and stifled by this tradition, SynBio must break with it. SynBio must offer an exit from the everlasting descriptive treadmill by asking if current information is already enough to enable design or redesign a life process, e.g., a regulatory circuit or a metabolic pathway. And if the built design fails, SynBio must ask why, and find what needs fixing.^1^ In short, SynBio’s engineering mindset can put a refreshingly sharp point on the quest for knowledge and target enquiry to the points where deep understanding is most lacking, and where getting it will have the greatest intellectual and societal payoffs–and be most fundable.

## Cells versus Circuit Boards

It is remarkable that so much emphasis has been placed on SynBio’s promise (typically illustrated with short-term experiments) and so little on the fundamental issues that need tackling to make *biology-as-engineering* a reality. It is damaging and risky for SynBio’s future that such key issues are often swept under the carpet for the sake of showing a rosy picture. Topping the issue list is the inherent ability of biological systems to mutate and evolve not only when subjected to changing environmental conditions but also merely via genetic drift.^14^ Another issue is that the performance of biological devices is characteristically context-dependent, which makes the claim of engineered orthogonality more like semantic wishful-thinking than a reality. Context-dependence leads to emergence of interactions and properties that are hard to predict from first principles. Finally, the physical stuff of biological matter is *soft*; its “hardware” comprises malleable and flexible parts, glues, phase separation, etc. These properties and their mechanistic details are scarcely found in human-engineered objects, which are made of *hard* components. Cells are not circuit boards. If we seek durability and predictability of SynBio constructs, we need to face these issues and to try to manage them instead of pretending they do not exist.^15^

## Hope versus Hype

Like any advance, SynBio evokes sincere evangelical zeal in its practitioners, which is good in itself and helps spread SynBio ideas and technology throughout the public and private sectors.^16^ Informed, data-driven forecasting of what SynBio advances could enable is likewise good and helpful. Diffusing advances and faithfully predicting their impacts fuel hope, a virtue we cannot afford to lose in facing what has been called a global polycrisis.^17^ Like any crisis, though, this one creates opportunities for merchants of false hope, or hype. Hype flourishes when disoriented, poorly informed populations *want to believe* in easy solutions to difficult problems; SynBio can be–and sometimes is–pushed in this way as a techno-fix in areas such as atmospheric CO_2_ drawdown,^18^ nitrogen pollution in agriculture,^19^ and green jet fuel production.^20^ It is not that SynBio cannot contribute to these areas; it can. The problem is one of claims for the scale and timeline of the contribution that do not follow the engineering practice of running the numbers to get rough but robust estimates of how much a SynBio-based project could possibly do and how fast,^21^ and then sticking to these estimates when advocating and publicizing the project. A habitual disregard of what is involved in scale-up is an Achilles’ heel of the whole field. It is one thing to have a smart genetic construct showing a spectacular phenotype in a Petri dish or small bioreactor for a short period of time but a very different thing to have the same at an industrial or even global scale^10^ and following rigorous implementation standards. Going from one dimension to the other makes the whole difference between a merely intellectual exercise and a truly transformative development.^22^ Alas, the conceptual excitement occasioned by SynBio has thus far been more supported than scale-up technologies–unfairly considered a lower-rank endeavor.^23^ The solution is just to learn from and to follow sound engineering practices and traditions. Not doing so, in the long term, only weakens public trust in bioscience and biotechnology.

## Tools, Toys, Training, and Translation

If SynBio is to make good on its promise of a new bioeconomy, it needs tools, a workforce trained to use them, and public acceptance.^24^ It is therefore essential to have a vibrant, outward-facing tool-building sector in universities and to train people to use today’s tools and to design and build tomorrow’s. Tool-building is thus part of training, and since such tools are being built–and published–by trainees they necessarily sometimes have the character of toys, i.e., devices to develop hands and minds. So far, so good. The problem comes when tool-building, detached from need and utility, proliferates to become an aim in itself and we get full toyshops but empty factories. Again, avoiding this problem just requires a dose of engineering realism,^25^ which in this case means (i) distinguishing tools developed mainly for training and research from ones that are seriously useful in practice or likely to be, and (ii) not overselling the former (see above). But these matters are not purely technical. Translating SynBio’s potential into reality requires acceptance of SynBio as a powerful technology for human, industrial, and societal progress, free of still-prevalent stigmas about genetically modified organisms. This calls for changing the metanarrative of modern biotechnology as an effort to subjugate the biological world to the goal of endless economic growth into a more positive one where SynBio enables a win-win partnership with nature.^26^ To this end, adequate scientific training should be matched by an effort to raise general biological literacy of the public and end-users as well as specialists.^27^

## Industrialization versus Trivialization?

A central promise of SynBio has been to “industrialize biology” by combining engineering principles such as computational design, standardization of parts, modularity, and abstraction with assembly line technology in the form of biofoundries.^24,28^ This combination is surely powerful and has been central to SynBio’s rollout. There are nevertheless–paradoxically–elements of both over- and under-promising here. The overpromising stems from the obvious fact that only a certain, rather narrow set of biological problems can be solved by industrialized implementation of the design-build-test-learn cycle. These problems relate predominantly to fermentations of some sort and to individual enzymes. While a biofoundry can speed up initial development of microbes for, e.g., microbiome engineering, the process is bottlenecked at the next step when the products have to prove their worth in farmers’ fields or patients’ guts.^29,30^ Similarly, plants and animals do not reproduce every 20 min or live in fermentation tanks. The under-promising stems from an almost trivializing tunnel-vision of what engineering is: it is not, and never has been, dependent on computational design and standardized parts, and even the most iconic SynBio successes owe more to old-fashioned trial-and-error tinkering than to an electronics-based vision of engineering.^31,32^ Overdominance of this chip-centric understanding of what SynBio is can limit conceptions of its future beyond the fermentor,^33,34^ which would be unfortunate. The essence of SynBio is to reimagine life processes and even life itself as they might be, subject to thermodynamic and kinetic constraints but not to the frozen accidents of evolutionary history.^6,35^ This can go beyond the typical efforts to apply to live objects the engineering principles used to construct complex devices by instead leveraging everything biological evolution has invented through a different logic for solving multiobjective optimization challenges.^36^

Lastly, we note that, while progress has been substantial,^37^ the above points on falling short of SynBio promises are uncomfortably close to those made in 2010 in a *Nature* News Feature titled ‘Five hard truths for synthetic biology’.^38^ This article included the quote “The field has had its hype phase. Now it needs to deliver”. A correctable reason for hype’s persistence is overspecialization, which is–and long has been–a feature of PhD training.^39^ The capacity to see beyond disciplinary boundaries to wider realities is part of engineering philosophy;^25^ it confers something close to a superpower in a specialist world^40^—and it can be taught.
